# Breastfeeding practices among women with diabetes: a cross-sectional study in a Tunisian hospital

**DOI:** 10.11604/pamj.2025.50.33.46153

**Published:** 2025-01-27

**Authors:** Chaima Sdiri, Haifa Abdesselem, Kamilia Ounaissa, Fatma Boukhayatia, Emna Bornaz, Asma Ben Brahim, Rim Yahyaoui, Chiraz Amrouche

**Affiliations:** 1 Department of Nutritional Diseases (D), National Institute of Nutrition and Food Technology, Tunis, Tunisia

**Keywords:** Breastfeeding, infant, newborn, diabetes mellitus

## Abstract

**Introduction:**

in literature, women with diabetes breastfeed less than other women of childbearing age. Our study aimed to determine the rates and modalities of breastfeeding in women with diabetes during six months post-delivery and to determine the factors associated with it.

**Methods:**

a prospective descriptive observational study was conducted in the Department of Nutritional Diseases “D” at the National Institute of Nutrition of Tunis over 18 months from January 2022 to June 2023. Breastfeeding in women with diabetes was assessed at one week (T1) in 78 patients, two months (T2) in 52 patients and six months (T3) post-delivery in 30 patients. Women with gestational diabetes and women who had an abortion, an intrauterine fetal death, or neonatal death, were excluded. Multivariable logistic regression analysis was performed to identify factors associated with breastfeeding.

**Results:**

the mean age of participants was 35.1±4.8 years. Most patients (82%) had type 2 diabetes. Exclusive breastfeeding rates were 40%, 44%, and 10% at T1, T2, and T3, respectively. Partial breastfeeding rates were 42%, 33%, and 40%, respectively. Multivariable analysis showed that the husband's encouragement (aOR: 2.45, 95% CI 1.34-4.12; p=0.003) was positively associated with breastfeeding, while prematurity (aOR: 0.56, 95% CI 0.34-0.92; p=0.02) was negatively associated. Other barriers included insufficient milk (p=0.001) and breast refusal (p < 0.001).

**Conclusion:**

factors promoting or hindering breastfeeding, such as family support and neonatal complications, should be systematically addressed in the postpartum care of women with diabetes.

## Introduction

Breastfeeding is recommended and encouraged for mothers, as it offers various health advantages for mothers and children [1-4]. Breast milk represents the best source of nourishment for the development of infants. Therefore, the World Health Organization (WHO) recommends exclusive breastfeeding at least six months [5]. However, despite strong evidence supporting WHO recommendations, there are significant fluctuations in the rates of breastfeeding, and multiple investigations have shown that women with diabetes, especially women with insulin-treated diabetes had the poorest outcomes concerning breastfeeding rates [6].

The existing literature had identified several factors associated with low breastfeeding adherence among women with diabetes, such as type of diabetes, antenatal care, prematurity, neonatal morbidity, high cesarean section rates, as well as young maternal age, multiparity, smoking and poor socio-economic conditions, etc. [6-8]. According to the 2018 Multiple Indicator Cluster Survey (MICS) in Tunisia, 92.2% of most recent live births in the last two years were breastfed, and 31.6% were breastfed within the first hour after birth. However, only 13.5% of infants were exclusively breastfed for the first six months of life [9]. However, national data focusing specifically on breastfeeding among women with diabetes are lacking. Given the increased maternal and neonatal risks associated with diabetes, understanding breastfeeding practices in this population is crucial to inform targeted interventions. This study aims to determine breastfeeding rates and practices among women with diabetes who consulted the Department of Nutritional Diseases (D) over one year and to identify factors associated with breastfeeding in this population.

## Methods

**Study design and setting:** a prospective descriptive observational study was conducted at the Nutrition Diseases Department “D” of the National Institute “Zouhaier Kallel” of Nutrition and Food Technology of Tunis. The study spanned 18 months, from January 2022 to June 2023, and included four successive consultations to gather comprehensive participant data.

**Study population:** the study targeted pregnant women with type 1 or type 2 diabetes, as well as women not previously diagnosed with diabetes but exhibiting a fasting blood glucose level of ≥1.26 g/dL and/or glycosylated hemoglobin (A1c) ≥6.5% in early pregnancy. Women with gestational diabetes and those who experienced abortion, intrauterine fetal death, or neonatal death were excluded. Eligible participants were identified through routine clinical visits during the study period.

**Data collection:** data were collected during four consultations. At the initial pregnancy consultation, information was gathered on general characteristics, obstetric history, diabetes characteristics, and breastfeeding knowledge. We assessed diabetes control by measuring the mean of the last three A1C.

Postpartum data were collected via phone within the first week (T1) to assess delivery methods, maternal and fetal complications, and breastfeeding practices. In the case of exclusive breastfeeding, we considered as criteria of effectiveness of breastfeeding: several feedings per day between 8 and 12, a change of heavy diapers 5 to 6 times per day and the presence of more than 3 soft yellowish granular stools per day.

Follow-up evaluations at two months (T2) and six months (T3) postpartum included assessments of socio-demographic factors such as return to work, socio-economic level and the role of the husband concerning breastfeeding, weight variations, glycemic control, hypoglycemic episodes, and breastfeeding modalities including number of feeds per day, use of a breast pump, obstacles to breastfeeding, diversification of the infant's diet and weight of the infant with calculation of the baby's weight gain. Instruments included structured questionnaires and clinical records, and measurements followed standard protocols.

**Definitions:** socio-economic level was classified based on annual per capita expenditure: low (≤1503 Tunisian dinars), medium (1504-3629 Tunisian dinars), and high (>3629 Tunisian dinars). Pre-gestational weight status was assessed using the WHO BMI classification, with obesity defined as a BMI ≥30 kg/m^2^. Hypoglycemia is defined as blood glucose levels below 0.7 g/l.

**Statistical analysis:** all statistical analyses were performed using the Statistical Package for Social Science, version 26.0. For univariate analysis, we used one-way ANOVA to compare means across more than two groups, Pearson's chi-square test or Fisher's exact test for percentage comparisons, and Pearson's correlation coefficient to assess linear relationships between continuous variables. Paired-sample t-tests were performed to compare pre-test and post-test means for the same individuals. For multivariate analysis, we applied stepwise descending logistic regression. Initially, all factors with p-values < 0.05 and those between 0.05 and 0.15 from the univariate analysis were included in the model. At each step, the least significant factor was removed until the final model was obtained. Adjusted odds ratios were calculated to determine the independent effect of each factor. For all analyses, a two-tailed p-value of 0.05 was considered significant.

**Ethical considerations:** the study protocol was conducted under the ethical principles outlined in the Declaration of Helsinki. Before enrollment, all participants provided informed consent after being fully informed of the study's purpose. The ethical committee of the National Institute “Zouhaier Kallel” of Nutrition and Food Technology of Tunis approved this study on May 26, 2022, under reference 14/2022.

## Results

**Figure 1:**
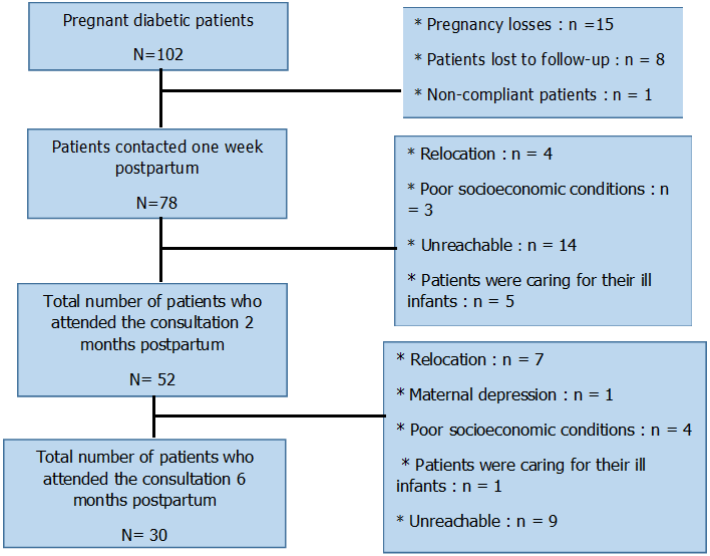
flowchart of the study population

**General characteristics of the study population:** the study included 102 participants, as shown in the flowchart (Figure 1). Demographic and general characteristics of the population at the first consultation in diabetology are summarized (Table 1). The average duration of diabetes was 3.6±2.9 and 13.6±7.3 years, for patients with type 2 diabetes and type 1 diabetes, respectively. The average A1C was 7.8±1.6%, with extremes ranging from 5.9 to 12%. Twenty-two percent of patients reported a hypoglycemic episode. Metformin was prescribed to 51% of patients with type 2 diabetes. Human insulin was the most prescribed insulin among patients with type 1 diabetes.

**Table 1:** study population characteristics at the first consultation in diabetology

Age (years)	35.1±4.8 Ext [23-44]
**Residence (%)**	
Urban	89.2
Rural	10.8
**Level of education (%)**	
Higher education	22.5
Secondary	43.1
Primary	27.5
Illiterate	6.9
**Socioeconomic level (%)**	
Low	28.4
Medium	69.6
High	2
**Type of diabetes (%)**	
Type 1	17.7
Type 2	53.9
Diabetes discovered at first trimester of pregnancy	28.4
**Comorbidities (%)**	
Hypertension	2.9
Hypothyroidism	6.9
Dyslipidemia	5.9
Anemia	2.9
Obesity	37.2
**Obstetric history**	
Average gravidity	3 [ext:1-8]
Average parity	1.52 [ext:0-4]

**Breastfeeding knowledge, practices and complications:** the survey revealed varying levels of knowledge and perceptions about breastfeeding among participants. Regarding the recommended duration of breastfeeding, 63.7% believed it should last 24 months, while smaller proportions thought it should be 6 months (10.7%) or 12 months (3.9%). Notably, 19.6% of participants had no idea about the recommended duration. Concerning the ideal breastfeeding position, the majority (71.6%) identified the correct position as holding the baby in the mother's arms, whereas 9.8% suggested the baby should lie in front of the mother, and 18.6% had no idea. When asked about the benefits of breastfeeding for the baby, 52.9% recognized its role in promoting good growth, 22.5% highlighted its importance for immunity, and only 1% mentioned the enhanced mother-baby relationship. However, 22.5% had no knowledge of its benefits. The benefits of breastfeeding for the mother were far less known, with 80.4% unable to identify any advantages. Among those who were aware, 19.6% correctly identified breastfeeding as offering protection against breast cancer. Regarding foods to avoid during breastfeeding, 88.2% of participants had no knowledge, while only 6.9% demonstrated true knowledge. The remaining respondents provided partially true knowledge (2.9%) or false knowledge (2%). Similarly, in terms of foods to prioritize during breastfeeding, 83.3% of participants had no idea, while 6.9% displayed accurate knowledge, 5.9% had partially true knowledge, and 3.9% provided incorrect answers. This analysis underscores significant gaps in breastfeeding-related knowledge among the surveyed population.

The study identified various maternal and neonatal complications associated with diabetes during pregnancy. Among maternal complications, preeclampsia was the most frequent, occurring in 11% of cases (n=9), followed by diabetic ketoacidosis (8%, n=6) and maternal hypoglycemia (4%, n=3). Postpartum depression was also noted in 4% of cases (n=3). Neonatal complications were significant, with neonatal respiratory distress being the most common (27%, n=21), followed by macrosomia (23%, n=18) and fetal hypoglycemia (22%, n=17). Other complications included neonatal jaundice (5%, n=4) and neonatal hypotrophy (4%, n=3). Additionally, 8% of neonates (n=6) required hospitalization in the neonatal intensive care unit. Fetal malformations were observed in 8% of cases (n=6), with specific anomalies including cardiac malformations (n=3), hydrocephalus (n=2), and renal malformations (n=1). These findings highlight the multifaceted risks associated with diabetes during pregnancy and its impact on both maternal and neonatal health. One hour after delivery, no breastfeeding was noted among patients included in the study. Breastfeeding rates and practices were observed to vary at one week, two months, and six months postpartum (Table 2).

**Table 2:** breastfeeding rates and practices at one week, two months and six months after delivery

	T1	T2	T3
Exclusive breastfeeding n (%)	31(40)	23(44)	3(10)
Partial breastfeeding n (%)	33(42)	17(33)	12(40)
Formula feeding n (%)	14(18)	12(23)	15(50)
Breast pump use n (%)	16 (21)	7 (14)	2 (7)
Average number of feedings per breast per day	7	6	2
Average number of feedings per bottle per day	4	3	4

T1: one week after childbirth; T2: two months after childbirth; T3: Six months after childbirth

**Factors associated with breastfeeding:** in T1, successful breastfeeding was associated with a residence with a small family (p=0.048), better glycemic balance (p=0.028), and vaginal delivery (p=0.002). Factors such as diabetic ketoacidosis (p=0.008), maternal hypoglycemia (p=0.035), prematurity (p=0.035), insufficient breast milk (p=0.001), breast pain (p=0.022), breast refusal (p=0.001), newborn hospitalization (p<0.001), and delayed lactation onset (p<0.001) were associated with breastfeeding failure. In T2 and T3: the husband's encouragement of breastfeeding increased the woman's adherence to breastfeeding (p<0.001 and p=0.001). Insufficient breast milk quantity (p=0.001 and p=0.004), breast refusal (p<0.001 and p=0.003), and bottle adherence (p<0.001 and p=0.01) were the main obstacles. Multivariate analysis identified the husband's encouragement for mother to breastfeed: OR=0.009, 95%CI 0.001-0.119; p<0.001 and prematurity: OR=6.068, 95%CI 1.324-27.802; p=0.020, as factors independently associated with breastfeeding (Table 3).

**Table 3:** factors associated with success or failure of breastfeeding one week, two months and six months after childbirth

				Unadjusted ORs (95% CI)	P-value	Adjusted ORs (95% CI)	P-value
**One week after childbirth**	Factors associated with successful breastfeeding	Living with a small family		1.27 (1.11−1.47)	**0.048**		
		Vaginal delivery		6.41 (2.12−19.33)	**0.002**	6.40 (2.13−19.34)	**0.002**
		Better glycemic control		2.27 (1.7-9.66)	**0.028**		
	Factors associated with breastfeeding failure	Diabetic ketoacidosis		6.14 (6.011-7.723)	**0.008**		
		Maternal hypoglycemia		0.14 (0.011-0.723)	**0.035**		
		Prematurity		3.57 (1.4 – 32.0)	**0.035**	6.068 (1.324–27.802)	**0.02**
		Insufficient breast milk		0.2 (0.0165–0.43)	**0.001**		
		Breast pain		2.29 (1.8–59.66)	**0.022**		
		Breast refusal		0.2 (0.0165–0.7)	**0.001**		
		Newborn hospitalization		0.0082 (0.0008 –0.087)	**<0.001**		
		Delayed lactation onset		20.94 (1.11–397.62)	**<0.001**		
	Factors that did not affect adherence to breastfeeding	Presence of family assistance		0.33 (0.07–1.46)	0.25		
		Socio-economic level		0.37 (0.01–1.39)	0.22		
		Breast pump use		6 (0.79–45.16)	0.08		
		Type of diabetes		4 (0.59–34.16)	0.22		
		Breastfeeding education		3.79 (1.89–7.70)	0.23		
**Two months after childbirth**	Factors associated with successful breastfeeding	Breastfeeding initiation during the first week after childbirth		1.6 (1.22–6.85)	**<0.001**		
		Higher knowledge levels about breastfeeding		0.29 (0.16–0.58)	**0.048**		
		Absence of returning to work		1.29 (1.13–1.57)	**0.007**		
		Husband's encouragement of breastfeeding		0.008 (0.001–0.117)	**<0.001**	0.009 (0.001–0.119)	**<0.001**
	Factors associated with breastfeeding failure	Insufficient breast milk quantity		2.15 (1.36–3.48)	**0.001**		
		Breast refusal		2.98 (2.25–3.89)	**<0.001**		
		Bottle adherence		0.4 (0.32–0.52)	**<0.001**		
	Factors that did not affect adherence to breastfeeding	Residence	0.92 (0.58–1.61)		0.73		
		Socio-economic level	0.96 (0.82–1.19)		0.19		
		Breast pump use	0.27 (0.12–1.92)		0.68		
		Mode of delivery	1.8 (0.78–5.35)		0.22		
		Type of diabetes	0.99 (0.82–1.39)		0.84		
		Glycemic control	0.48 (0.39–1.48)		0.41		
**Six months after childbirth**	Factors associated with successful breastfeeding	Husband's encouragement of breastfeeding	2.17 (1.4–3.34)		**0.001**		
	Factors associated with breastfeeding failure	Insufficient breast milk quantity	0.8 (0.68–0.94)		**0.004**		
		Breast refusal	0.26 (0.11–0.63)		**0.003**		
		Bottle adherence	1.43 (1.01–2.35)		**0.01**		
	Factors that did not affect adherence to breastfeeding	Residence	0.65 (0.28–1.47		0.1		
		Socio-economic level	0.65 (0.25–1.68)		0.44		
		Return to work	0.93 (0.6–1.56)		0.52		
		Breast pump use	1.09 (0.89–1.32)		0.19		
		Type of diabetes	0.26 (0.05–1.79)		0.75		
		Glycemic control	0.97 (0.83–1.18)		0.14		

## Discussion

Breastfeeding offers numerous benefits for both mothers and their babies. However, diabetes may complicate the initiation and continuation of breastfeeding. In this study, we investigated the rates and modalities of breastfeeding in a population of women with diabetes during the first six postpartum months and the associated factors.

This study reported no skin-to-skin contact and no breastfeeding was initiated within one hour after delivery. However, the MICS conducted in Tunisia in 2018, showed, a rate of skin-to-skin contact of 13% and a prevalence of breastfeeding within one hour after delivery of 31.6%, among 1,230 births [9]. Similarly, the study conducted by Soltani and Arden, in 2009, showed a breastfeeding rate at delivery of 66.7% in patients with type 1 diabetes and 81.8% in those with type 2 diabetes [10]. This difference between studies could be explained by the mandatory medical monitoring of babies immediately after birth in the nursery or neonatal units, especially those born to diabetic mothers, which may reduce the time of contact between the mother and the newborn.

One week after childbirth, we found exclusive and partial breastfeeding rates of 40% and 42%, respectively. Our results were similar to those found in the study conducted by Soltani and Arden [10]. The Cordero *et al*. study including 392 patients with diabetes, showed an exclusive breastfeeding rate of 3% and a partial breastfeeding rate of 35% among newborns who were kept in the nursery after birth. For babies admitted to the neonatal intensive care unit, 13% were exclusively breastfed, and 23% were partially breastfed [11]. Disparities in results could be partially attributed to the varying rates of maternal-fetal complications in previous studies, which may have extended the mother's recovery time and prolonged her separation from the baby. Our results showed that more than half of the patients had an A1c level >7% and that women who breastfed had a lower A1c level than those who did not breastfeed (p=0.028). These findings are consistent with the literature. Indeed, some studies have demonstrated a delay in lactogenesis in women with diabetes with poor glycemic control [12,13].

At two months postpartum, we found exclusive and partial breastfeeding rates of 44% and 33%, respectively. A study conducted in Sweden between 2007 and 2009 showed that 80.7% of women with type 1 diabetes were breastfeeding their babies at two months postpartum, with an exclusive breastfeeding rate of 80% [8]. Another study conducted in the same country between 2011 and 2014 demonstrated that the prevalence of breastfeeding had not changed (78.6%) [14]. These findings were corroborated by Ringholm *et al*., who showed that at this age, 73% of women with type 1 diabetes included in the study were breastfeeding their babies [15]. This difference could be explained by the high breastfeeding rate in this country, which exceeded 90% among non-diabetic women. In Italy, women with diabetes breastfed slightly less (66%) according to the Riviello *et al*. study conducted between 2006 and 2008 [16]. Our results showed that the initiation of breastfeeding at T1 was associated with continued breastfeeding up to two months postpartum (p<0.001). This association was confirmed by the study of Cordero *et al*. which demonstrated that initiation of breastfeeding at the time of hospital discharge was an important predictor of continued breastfeeding at two and four months among non-diabetic women and women with type 1 diabetes [11].

Our results showed exclusive and partial breastfeeding rates at six months postpartum of 10% and 40%, respectively. However, the MICS survey showed an exclusive breastfeeding rate in Tunisia of 13% [9]. The literature reports a wide range of results across different studies. Linden *et al*. found that 53% of women with type 1 diabetes in Sweden were breastfeeding their babies between 2011 and 2014 [14]. It is worth noting that the general Swedish population had one of the highest breastfeeding rates at six months globally, with 65% of infants being breastfed (any type of breastfeeding) and 10% exclusively breastfed [17]. In Finland, a study reported a very low breastfeeding rate among infants of mothers with type 1 diabetes, with only 1.4% exclusively breastfed [18]. A study conducted in Saudi Arabia in 2019 showed that 29.1% of diabetic patients exclusively breastfeed their babies for up to six months of age [19]. These disparities in results were due to significant differences in sample sizes across studies and various socio-cultural behaviors from one region to another.

In our study, the most reported barriers to breastfeeding by patients were insufficient breast milk production or a complete absence of milk (p=0.004), baby's refusal of the breast (p=0.003), and baby's preference for the bottle (p=0.01). These findings are consistent with the results of a survey conducted in England that aimed to investigate the reasons why women with diabetes had stopped breastfeeding. The most reported reasons were lack of milk (25%), problems with latching (13%), time constraints (13%), returning to work (11%), maternal health conditions (9%), convenience (5%), and natural weaning progression (22%) [20].

Overall, the study has several strengths and limitations which were considered when interpreting the findings. To our knowledge, it is the first Tunisian study focused on breastfeeding in women with diabetes. Its prospective design allowed us to follow women with diabetes from their first diabetology consultation during pregnancy up to six months postpartum. However, a significant number of patients were lost to follow-up: only 52 were followed until two months postpartum, and just 30 completed the study. This may be attributed to challenges in finding childcare during appointments and the impact of the fifth wave of COVID-19, which hindered hospital visits. Additionally, the study did not assess the nutritional intake of breastfeeding women, which could influence glycemic control and breastfeeding continuation.

## Conclusion

The results showed that in T1, successful breastfeeding was associated with a residence with a small family, better glycemic control and vaginal delivery. Factors such as diabetic ketoacidosis, maternal hypoglycemia, prematurity, insufficient breast milk, breast pain, breast refusal, newborn hospitalization and delayed lactation onset, were associated with breastfeeding failure. In T2 and T3, husband's encouragement of breastfeeding increased women's adherence to breastfeeding. Insufficient breast milk quantity, breast refusal and bottle adherence were the main obstacles. Multivariate analysis identified the husband's encouragement of breastfeeding and prematurity as factors independently associated with breastfeeding. Breastfeeding should be an absolute priority. We suggest promoting skin-to-skin contact and emphasizing the role of the surrounding environment, particularly the husband's role.

### What is known about this topic

- Breastfeeding rates are lower in women with diabetes due to medical and psychosocial barriers;
- Diabetic women face challenges like delayed lactogenesis, milk production concerns, and glycemic control;
- Diabetes-related complications may negatively impact breastfeeding initiation and duration.

### What this study adds

- Identifies delayed lactogenesis, insufficient milk production, and neonatal hospitalization as major barriers to breastfeeding among diabetic women;
- Highlights the protective role of partner and family support in promoting breastfeeding continuation;
- Emphasizes the importance of early breastfeeding initiation, rooming-in, skin-to-skin contact, and lactation support for diabetic mothers.
